# A single nucleotide mutation in *GID1c* disrupts its interaction with DELLA1 and causes a GA‐insensitive dwarf phenotype in peach

**DOI:** 10.1111/pbi.13094

**Published:** 2019-03-06

**Authors:** Jun Cheng, Mengmeng Zhang, Bin Tan, Yajun Jiang, Xianbo Zheng, Xia Ye, Zijing Guo, Tingting Xiong, Wei Wang, Jidong Li, Jiancan Feng

**Affiliations:** ^1^ College of Horticulture Henan Agricultural University Zhengzhou China

**Keywords:** peach, dwarfism, gibberellic acid, GID1c, DELLA protein, transcriptome

## Abstract

Plant stature is one important factor that affects the productivity of peach orchards. However, little is known about the molecular mechanism(s) underlying the dwarf phenotype of peach tree. Here, we report a dwarfing mechanism in the peach cv. FenHuaShouXingTao (FHSXT). The dwarf phenotype of ‘FHSXT’ was caused by shorter cell length compared to the standard cv. QiuMiHong (QMH). ‘FHSXT’ contained higher endogenous GA levels than did ‘QMH’ and did not response to exogenous GA treatment (internode elongation). These results indicated that ‘FHSXT’ is a GA‐insensitive dwarf mutant. A dwarf phenotype‐related single nucleotide mutation in the gibberellic acid receptor GID1 was identified in ‘FHSXT’ (*GID1c* ^*S191F*^), which was also cosegregated with dwarf phenotype in 30 tested cultivars. GID1c^S191F^ was unable to interact with the growth‐repressor DELLA1 even in the presence of GA. ‘FHSXT’ accumulated a higher level of DELLA1, the degradation of which is normally induced by its interaction with GID1. The DELLA1 protein level was almost undetectable in ‘QMH’, but not reduced in ‘FHSXT’ after GA_3_ treatment. Our results suggested that a nonsynonymous single nucleotide mutation in *GID1c* disrupts its interaction with DELLA1 resulting in a GA‐insensitive dwarf phenotype in peach.

## Introduction

The cultivation of dwarf fruit trees is widely used in modern production of many fruits, such as apple, sweet cherry and peach, since it reduces the length of the juvenile period, results in higher quality and yield and allows for efficient mechanized management (Lang *et al*., 2001; Smolka *et al*., 2010). In peach production, horticultural practices such as pruning and spraying growth retardant are usually used to constrain tree growth, which is costly in terms of materials, time and labour. Therefore, it is important to find a way of controlling tree size efficiently. With the rapid development of genome‐editing techniques, creating dwarfed cultivars through genetic modification may prove more efficient than traditional breeding technology. However, the molecular mechanism(s) of dwarfism in peach is unclear. Therefore, research on the underlying mechanisms that yield smaller statured peach trees will be helpful for breeding peach cultivars suitable to dwarfed culture.

The mechanisms conveying dwarfed statures have been extensively researched in model plants. Gibberellic acids (GAs) are diterpenoid plant hormones that have multiple biological functions, especially the promotion of stem elongation (Bömke and Tudzynski, 2009). In plants, GA biosynthesis is catalyzed by six key enzymes, including ent‐copalyl diphosphate synthase (CPS), ent‐kaurene synthase (KS), ent‐kaurene oxidase (KO), entkaurenoic acid oxidase (KAO), GA 20‐oxidase (GA20ox), GA 3‐oxidase (GA3ox) and its deactivation is catalyzed by GA 2‐oxidase (GA2ox) (Yamaguchi, 2008). GAs promote stem elongation by stimulating the degradation of the growth‐repressing DELLA proteins. The degradation of DELLA is triggered by the formation of a GA‐GID1‐DELLA complex, which is then identified by a specific ubiquitin E3 ligase complex (SCF^SLY1/GID2^) for polyubiquitination, which marks the DELLA proteins for degradation by the 26S proteasome (Sun, 2010). Mutations in GA biosynthesis genes, such as KO (Itoh *et al*., 2004), GA20ox (Oikawa *et al*., 2004; Plackett *et al*., 2012) and GA3ox (Chen *et al*., 2014; Itoh *et al*., 2001), cause dwarfism by decreasing the endogenous levels of GA, leading to accumulation of DELLA proteins, which then restrict internode elongation. GA biosynthesis can also be influenced by other molecular mechanisms. OsEATB negatively regulates internode length in rice by repressing the transcript level of *KS* (Qi *et al*., 2011). Overexpression of *OsWOX3A* down‐regulates GA biosynthesis by suppressing the transcription of *KAO*, resulting in shorter internode length (Cho *et al*., 2016). The expression of *KO* is down‐regulated in the *gdd1* mutant, which causes dwarfism of rice (Li *et al*., 2011). In addition, mutations in the GA signalling pathway can alter plant stature. Apart from the down‐regulation of GA3ox and DWF4 (a brassinosteroid biosynthetic gene), the NAC transcription factor JUB1 can directly activate the transcription of the growth‐repressor *DELLA* (Shahnejat‐Bushehri *et al*., 2016). The protein phosphatase TOPP4 acts as a positive regulator in the GA signallling pathway by promoting the GA‐induced degradation of DELLA proteins (Qin *et al*., 2014).

Brassinosteroids (BRs) and auxin play positive roles in regulation of plant stature. BRZ, a triazole compound that specifically blocks brassinolide biosynthesis, causes dwarf phenotypes similar to those of BR‐deficient mutants (Wang *et al*., 2002). PP2A activates brassinosteroid‐responsive gene expression and plant growth by dephosphorylating BZR1 (Tang *et al*., 2011). Hypocotyl elongation is promoted by treatment with the artificial auxin picloram (Oh *et al*., 2014). The auxin response factor ARF6 and its close homolog ARF8 redundantly regulate hypocotyl elongation in Arabidopsis (Nagpal *et al*., 2005). Additionally, environmental signals, including light and temperature, modulate plant stature (Gangappa and Kumar, 2017).

These environmental and endogenous signals, including light, temperature, BRs, gibberellic acid (GA) and auxin, regulate stem elongation largely by promoting cell elongation (Bai *et al*., 2012a; Oh *et al*., 2014; Gangappa and Kumar, 2017). The interactions between every signal create a balanced growth, with the DELLA proteins having a pivotal role in regulating multiple hormone signals (Davière and Achard, 2016). Molecular and physiological studies have demonstrated that DELLA proteins interact with transcription factors, such as BZR1, PIF4 and ARF6 and inhibit their transcriptional activity (de Lucas *et al*., 2008; Feng *et al*., 2008; Bai *et al*., 2012a,b; Oh *et al*., 2014). In addition, strigolactone (SL) biosynthesis could be regulated by gibberellin signalling. The epi‐5DS (one of SL) is undetectable in the constitutive GA response mutant *slr1‐5* (a DELLA protein; Ito *et al*., 2017). SLs are a group of plant compounds involved in the coordination of plant growth via the regulation of shoot branching (Brewer *et al*., 2016; Ito *et al*., 2017). In SL mutants, a reduction in plant height may be an indirect effect of increased tillering or branching, causing a redirection of resources towards increasing branch number instead of stem elongation (Wang *et al*., 2018). It is possible that GA could regulate shoot branching by modulating SLs biosynthesis.

Peach [*Prunus persica* L. (Batsch)], a member of the Rosaceae family, is the third most important deciduous fruit tree worldwide. Breeding dwarfed peach cultivars is an effective way to reduce the cost of materials, time and labour and to increase productivity. In a peach cultivar with a recessive brachytic dwarfism trait, fine mapping identified the gibberellic acid receptor GID1c, which suggested that modification of GID1c expression could provide a rational approach to controlling tree size without impairing fruit development (Hollender *et al*., 2016). Cantín *et al*. (2018) reported a new SNP in *GID1c* gene is correlated with the phenotype of brachytic dwarfng in peach. However, the mechanism through which GID1c regulates the stature of peach tree is still unclear. In this study, a new mutation in GID1c in the dwarf cultivar ‘FHSXT’ was identified, and the molecular mechanism underlying the dwarf phenotype was investigated.

## Results

### Phenotypic analysis of the peach cultivars ‘FHSXT’ and ‘QMH’

**Figure 1:**
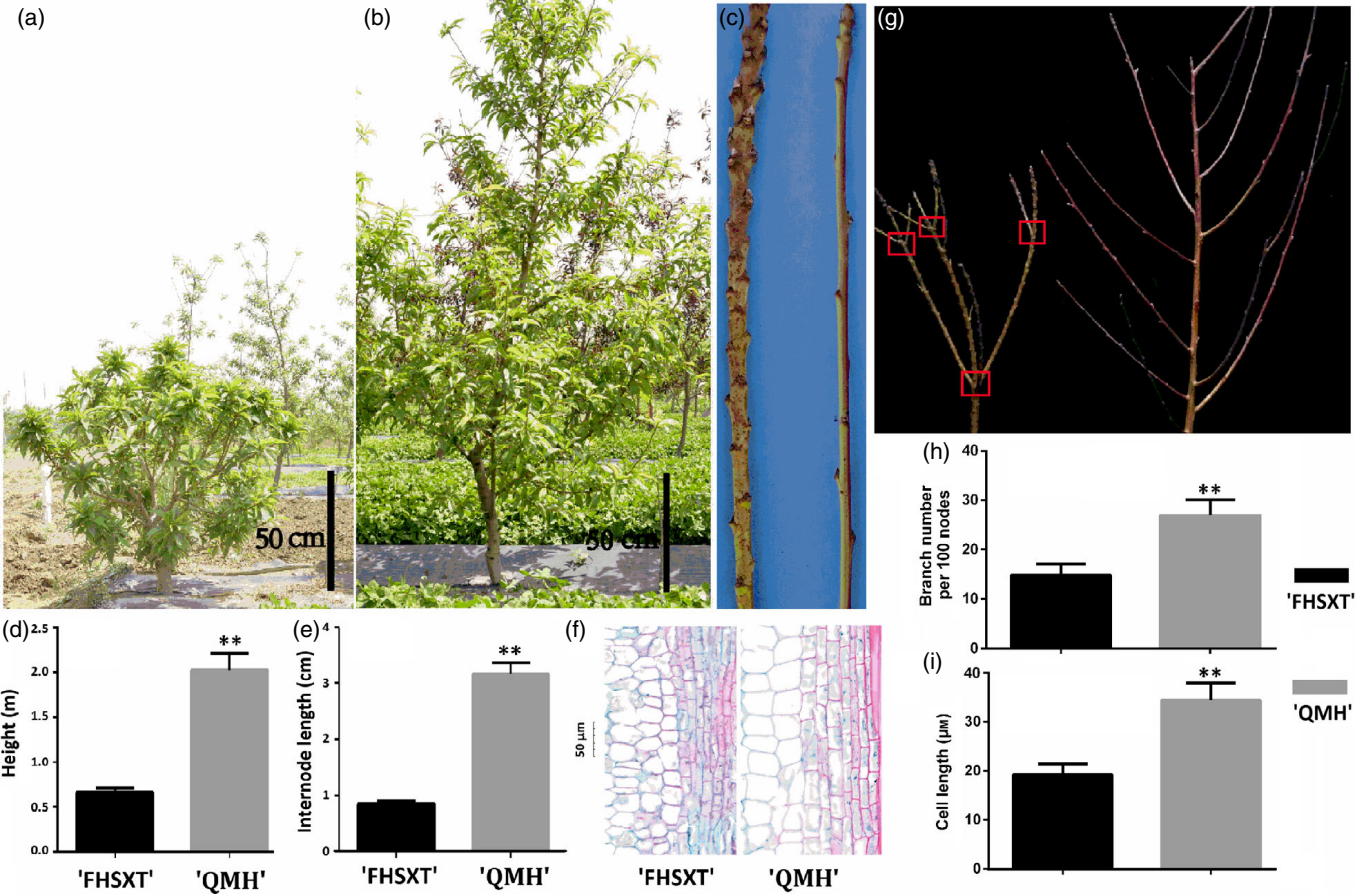
The phenotype of ‘FHSXT’ and ‘QMH’. Two‐year‐old dwarf ‘FHSXT’ (a) and 2‐year‐old standard ‘QMH’ (b) peach tree. (c) Branch from a ‘FHSXT’ and ‘QMH’ showing the internode length of the two cultivars. (d) Average height and (e) internode length of ‘FHSXT’ and ‘QMH’. (f) The cell length in semi‐lignified stems from ‘FHSXT’ and ‘QMH’. (g) Branch numbers in ‘FHSXT’ and ‘QMH’. The red frame highlights the position of branch outgrowth from the main stem. (h) Average numbers of brancher of the two cultivars. (I) Average length of parenchyma cell in stems from ‘FHSXT’ and ‘QMH’. The values represent the average of five biological replicates and error bars represent the standard deviation. ** indicate significant difference at *P *<* *0.001 between the two cultivars as determined by Student's *t* test.

‘FenHuaShouXingTao’ is a cultivar of *P. persica* with a dwarf phenotype, and ‘QMH’ is a standard cultivar selected from the population of ‘DaJiuBao’ × ‘Guangtao 5’. ‘FHSXT’ averaged 0.8 m in height, whereas ‘QMH’ trees averaged 2.2 m (Figure 1a, b & d). Average internode length of ‘FHSXT’ was 72% shorter than that of ‘QMH’ (Figure 1c & e). In paraffin section, the cell length was significantly shorter in ‘FHSXT’ than those of ‘QMH’ (Figure 1f & i). These results strongly suggested that this reduced cell length is the main reason underling the reduced internode length, which accounts for the dwarfed phenotype of ‘FHSXT’.

In this study, the number of branches was also analysed between ‘FHSXT’ and ‘QMH’. The number of sylleptic shoots from the lateral bud of proleptic shoot was statistically analysed. Within the same number of nodes, the number of branches was significantly reduced in ‘FHSXT’ compared to ‘QMH’. The outgrowth of lateral or vegetative buds near to the apex is externally similar to the forked branching character reported by Hu and Scorza (2009) (Figure 1g). In addition, the leaf length was longer in ‘FHSXT’ than in ‘QMH’ (Fig. S1).

### ‘FHSXT’ is a GA‐insensitive mutant

**Figure 2:**
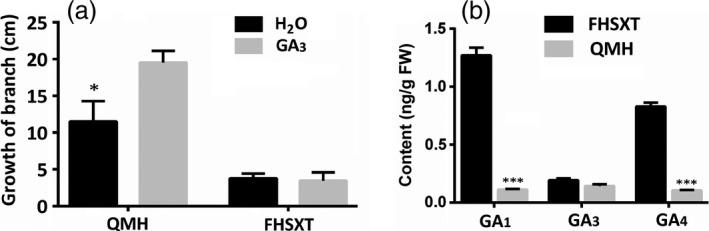
‘FHSXT’ is a GA‐insensitive mutant. (a) The growth of branches of ‘QMH’ and ‘FHSXT’ after weeks of GA_3_ treatment. (b) The contents of GA1, GA3 and GA4 in shoot tips from ‘FHSXT’ and ‘QMH’. The values represent the average of three biological replicates and error bars represent the standard deviation. * and *** indicate significant difference at *P *<* *0.05 and *P *<* *0.001 between the two cultivars as determined by Student's *t* test.

GA is one of the important hormone regulating stem elongation. The response of internode growth to exogenous GA treatment was assessed. Exogenous GA treatment significantly promoted stem elongation in ‘QMH’, but did not affect the internode length in ‘FHSXT’ (Figure 2a). The concentrations of endogenous GAs in the shoot tips of ‘FHSXT’ and ‘QMH’ were compared using HPLC‐MS‐MS analysis. The concentration of GA_1_, GA_3_ and GA_4_, which are the most common active forms (Hedden and Thomas, 2012), was determined. No significant difference in the concentration of GA_3_ was detected between the two peach cultivars, while the concentration of GA_1_ (1.27 ng/g) and GA_4_ (0.83 ng/g) in ‘FHSXT’ were significantly higher than those in ‘QMH’ (both at 0.11 ng/g; Figure 2b). These results strongly suggested that ‘FHSXT’ is a GA‐insensitive mutant.

### Differentially expressed genes (DEGs) between ‘FHSXT’ and ‘QMH’ shoot tip transcriptomes

To dissect the molecular mechanism behind the dwarfed phenotype of ‘FHSXT’, the total transcriptome of shoot tips from ‘FHSXT’ and ‘QMH’ was analysed using RNA‐seq. Three biological replicates for each cultivar were made into six cDNA libraries. For each replicate, about 6.58 of Gb clean bases were generated using the BGISEQ‐500 Platform. More than 94% of the clean reads were successfully mapped to the peach reference genome (Prunus_persica_v2.0) and the gene mapping ratio was more than 90% in each sample. Hierarchical clustering of gene expression divided six samples into two groups (Fig. S2a). Pearson correlation values between six samples were calculated with correlation coefficient (*r*) of biological replicates at more than 0.95 (Fig. S2b). These results suggested that the RNA‐seq data of the biological replicates are suitable for the following integrative analysis.

To identify differentially expressed genes (DEGs) between ‘FHSXT’ and ‘QMH’, a relatively strict criterion [fold change ≥ 2 and false discovery rate (FDR) ≤ 0.05] was employed. A total of 2198 significant DEGs were discovered, comprising 1335 up‐regulated and 863 down‐regulated DEGs in ‘FHSXT’ compared to ‘QMH’. Detailed information for all significantly DEGs is listed in Table S3.

### Up‐regulation of genes involved in GA synthesis and signalling in the dwarf ‘FHSXT’

**Figure 3:**
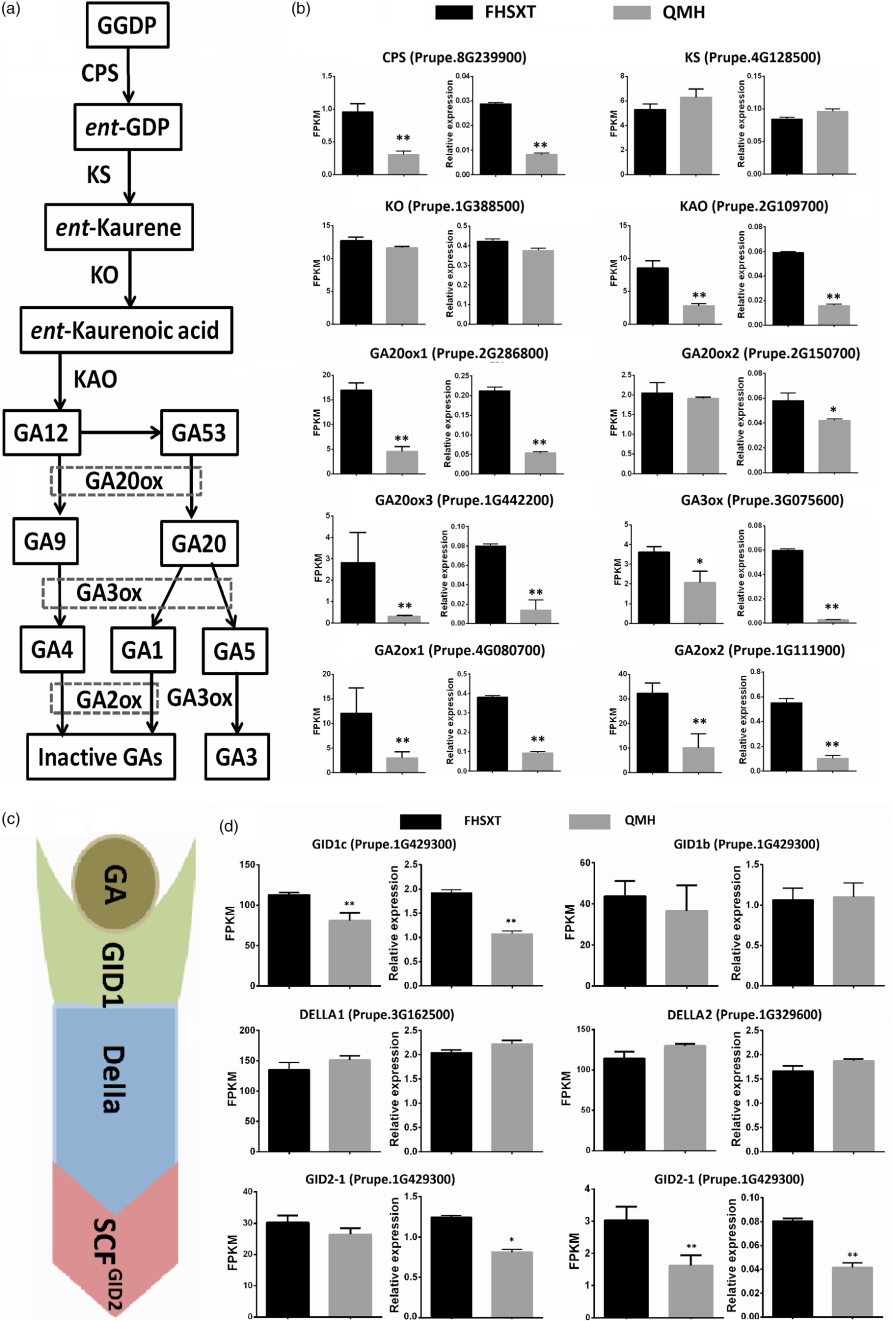
Transcriptional levels of GAs metabolic genes and signaling pathway genes in shoot tips from ‘FHSXT’ and ‘QMH’. (a) Schematic representations of the GA biosynthesis pathway. (b) The FPKM and relative transcript levels of the detected GA biosynthesis and catabolism genes. (c) Schematic representations of the GA signalling pathway. (d) The FPKM and relative transcript levels of the detected GA signalling pathway genes. Gene ID is shown next to the gene name. The values represent the average of three biological replicates and error bars represent the standard deviation. * and ** indicate significant difference at *P *<* *0.05 and *P *<* *0.01 between the two cultivars as determined by Student's *t* test.

It is well known that GA plays an important role in stem elongation, so the transcriptional levels of genes involved in GA metabolic and signalling pathway were analysed. GA is biosynthesized by six key enzymes, namely CPS, KS, KO, KAO, GA20ox, GA3ox and is deactivated by GA2ox (Figure 3a). Five biosynthetic genes, *CPS*,* KAO*,* GA20ox1*,* GA20ox3* and *GA3ox1*, were significantly up‐regulated in the dwarf ‘FHSXT’ (Figure 3b). Their transcript levels in ‘FHSXT’ were at least 1.7‐fold higher compared to in ‘QMH’, with *GA20ox3* showing the highest fold changes (8.9‐fold). This indicated that GAs were highly synthesized in ‘FHSXT’. *GA2ox1* and *GA2ox2*, responsible for deactivation of GAs, were also highly transcribed in ‘FHSXT’. In Arabidopsis and tomato, the treatment of exogenous GAs could activate the transcription of *GA2ox* (Livne *et al*., 2015). This further implied that endogenous GAs were highly accumulated in ‘FHSXT’.

The transcript level of GA signalling components, including the GA receptor1 (GID1), the F‐box protein (GID2) as well as the DELLA proteins, were also analysed (Figure 3c). Two *DELLA* genes and two *GID2* genes have been identified in the peach genome, but only *GID2‐2* showed a significant difference in transcript levels between ‘FHSXT’ and ‘QMH’. The peach genome contains two *GID1* genes (*GID1b* and *GID1c*) and *GID1c* was significantly up‐regulated in ‘FHSXT’ compared to ‘QMH’ (Figure 3d). Taken together, these results suggested that the changes in transcript level of genes involved in GA synthesis and signalling were not the reason for the dwarf phenotype of ‘FHSXT’.

### A nonsynonymous single nucleotide mutation in *GID1c* is related to the dwarf phenotype of ‘FHSXT’

**Figure 4:**
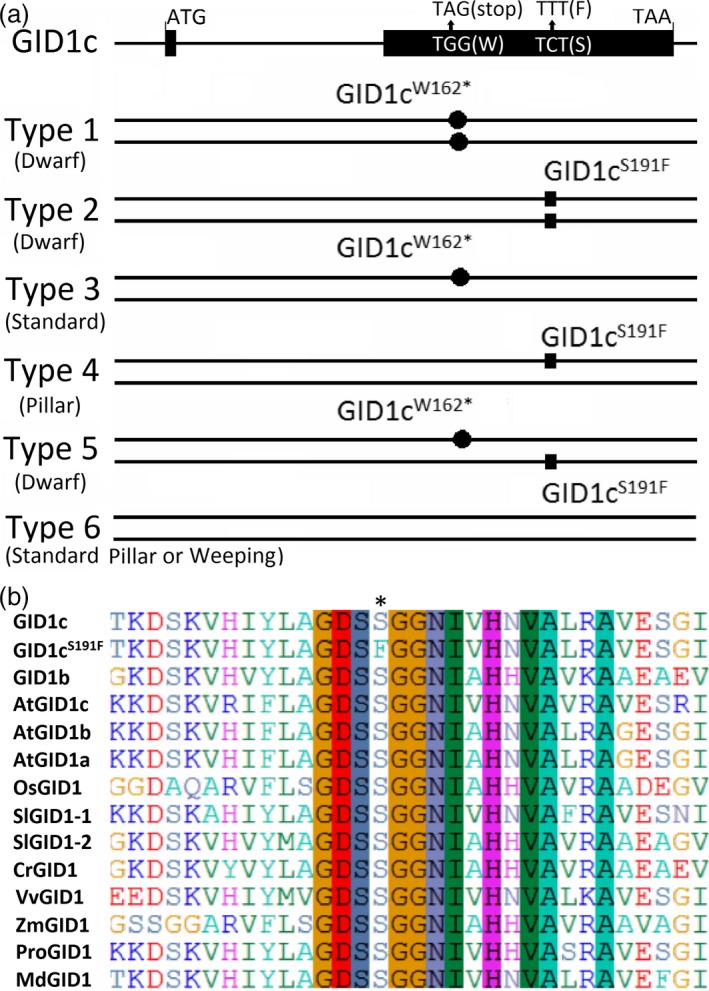
A nonsynonymous SNP in GID1c was related to the dwarf phenotype of ‘FHSXT’. (a) Based on only two SNPs in *GID1c*, 30 peach cultivars were divided into six types. Type 1 contains 3 dwarf cultivars ‘ZhongAi45’, ‘AiLiHong’ and ‘AiLiMi’ that carry the homozygous alleles *GID1c* ^W162*^; Type 2 contains eight dwarf cultivars ‘ShouBai’, ‘ShouFeng’, ‘DanShouHong’, ‘DanShouFeng’, ‘BaiShouXing’, ‘HongShouXing’, ‘Q37’ and ‘FHSXT’ that carry the homozygous alleles *GID1c* ^*S191F*^. Type 3 and Type 4 included one standard cultivars ‘Ke+Shi’ and the pillar cultivar ‘ZhaoShouFeng’ that carry the heterozygous alleles *GID1c* ^W162*^/*GID1c* or *GID1c* ^*S191F*^/*GID1c*, respectively. Type 5 included one dwarf cultivars ‘ZhongAi33’ that carry the heterozygous alleles *GID1c* ^W162*^/*GID1c* ^*S191F*^. Type 6 included the remaining the cultivars with normal plant height that carry the homozygous alleles *GID1c*. (b) The SNP from C to T in the coding sequence of *GID1c* caused conversion of S residue 191 to a F residue, which is located in the conserved motif ‘GDSSG’. * highlights the mutated animo acid.

The sequences of *GID1c* from ‘FHSXT’ and ‘QMH’ were analysed using GID1c‐SNP primers (Table S2). A single base pair mutation from C to T in the coding sequence of *GID1c* was identified in ‘FHSXT’, which causes conversion of serine (S) 191 to a phenylalanine (F; this allele was named *GID1c* ^*S191F*^). The coding sequences of *GID1c* from 30 cultivars with different tree growth habits were analysed. Only two kinds of SNPs were identified. The correlation between the SNPs in *GID1c* and dwarf phenotype was analysed. The 30 peach cultivars were divided into six types based on the presence of the two SNPs (Figure 4a). Type 1 included three dwarf cultivars (‘ZhongAi45’, ‘AiLiHong’, ‘AiLiMi’) and contained the alleles *GID1c* ^W162*^/*GID1c* ^W162*^ [the allele *GID1c* ^W162*^ was caused by a non‐sense mutation in *GID1c* and reported by Hollender *et al*. (2016)]. Type 2 included eight dwarf cultivars (‘ShouBai’, ‘ShouFeng’, ‘DanShouHong’, ‘DanShouFeng’, ‘BaiShouXing’, ‘HongShouXing’, ‘Q37’, ‘FHSXT’) and contained the alleles *GID1c* ^*S191F*^ */GID1c* ^*S191F*^. Type 3 included a standard cultivar (‘Ke+Shi’) and contained the alleles *GID1c* ^W162*^/*GID1c*. Type 4 included the pillar cultivar (‘ZhaoShouFeng’) and contained the alleles *GID1c* ^*S191F*^/*GID1c*. Type 5 included the dwarf cultivar ‘ZhongAi33’ and contained the alleles *GID1c* ^W162*^/*GID1c* ^*S191F*^. Type 6 included the remaining cultivars presenting standard, pillar or weeping growth habit and contained the alleles *GID1c*/*GID1c*. When aligned with GID1 from different species, the conversion of S to F occurred in the conserved motif GDSSG (Fig. 4b). These results showed a closed relationship between the two SNPs and the dwarf phenotype in peach.

### GID1c^S191F^ was unable to interact with DELLA1

**Figure 5:**
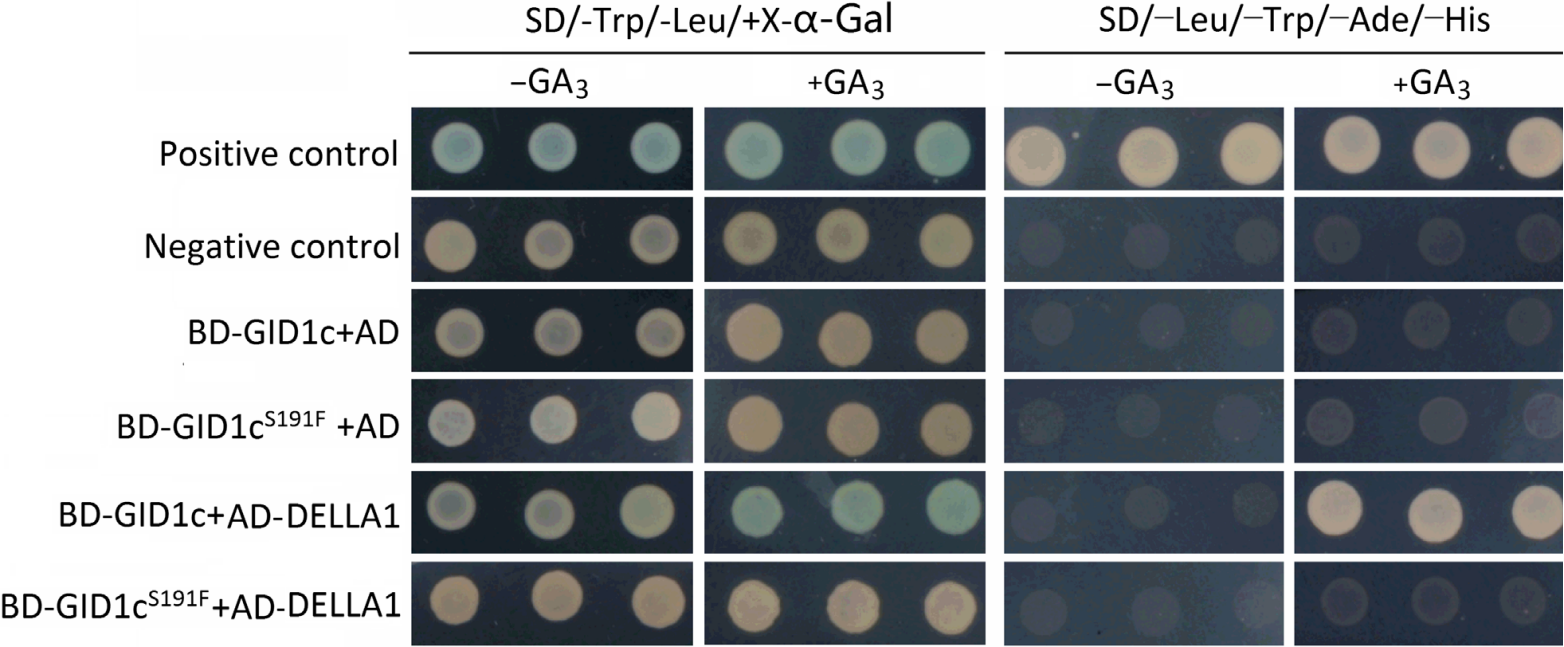
Interacion Analysis of GID1c and GID1c^S191F^ with DELLA1. Y2H assay using full‐length GID1c and GID1c^S191F^ as baits and the full‐length SLR1 as prey in the presence and absence of 10^−4^ m GA_3_. The Y2H assay was performed on SD/‐Trp/‐Leu/X‐α‐Gal and Top: SD/‐Trp/‐Leu/‐Ade/‐His.

The capabilities of GID1c and GID1c^S191F^ to interact with DELLA were analysed in the Y2H system (Figure 5). Two DELLA proteins, identified in the peach genome based on the sequence similarity, have the typical DELLA and VHYNP domain (Fig. S3). GID1c interacted with DELLA1 in yeast cells in the presence of GA_3_, but not in GA_3_‐free medium, while GID1c^S191F^ could not interact with DELLA1 in the presence or absence GA_3_. Neither GID1c nor GID1c^S191F^ showed an interaction with DELLA2 in the presence or absence of GA_3_ (Fig. S4). This result suggested that the S191F mutation in GID1c abolished the interaction of GID1c with DELLA1.

### DELLA1 protein was highly accumulated in ‘FHSXT’

**Figure 6:**
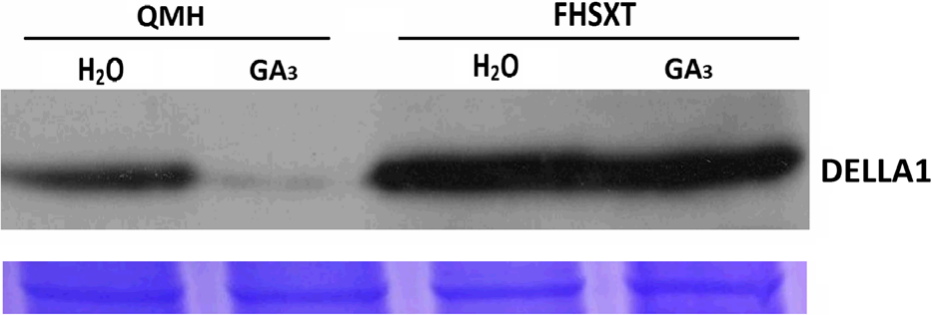
DELLA1 protein levels in shoot tips following treatment with GA. ‘QMH’ and ‘FHSXT’ plants were treated with H_2_O or 150 mg/L GA_3_ for 3 h. Coomassie brilliant blue‐staining the rubisco small subunit (RbcS) protein was used as a loading control.

The level of the DELLA1 protein was detected in the two cultivars via Western Blot (Figure 6). Compared with ‘FHSXT’, ‘QMH’ contains a moderate amount of DELLA1 proteins. After 3 h of GA treatment, the DELLA1 protein in ‘QMH’ was difficult to detect, but the DELLA1 protein in ‘FHSXT’ was not degraded compared with control. These results strongly suggested that the DELLA1 protein was degraded in ‘QMH’ in response to GA_3_ treatment.

### Analysing the genes regulating branch number

**Figure 7:**
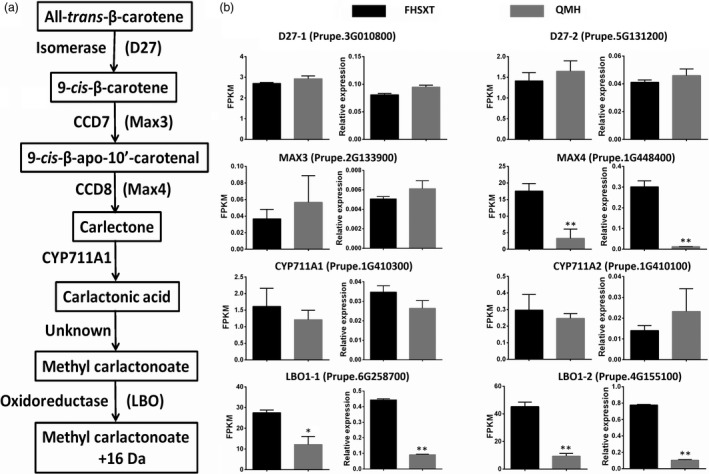
Transcriptional levels of SLs biosynthesis pathway genes in shoot tips from ‘FHSXT’ and ‘QMH’. (a) Schematic representations of the SLs biosynthesis pathway. (b) The FPKM and relative transcript levels of the detected SLs biosynthesis and catabolism genes. The values represent the average of three biological replicates and error bars represent the standard deviation. * and ** indicate significant difference at *P *<* *0.05 and *P *<* *0.01 between the two cultivars as determined by Student's *t* test.

The dwarf phenotype of ‘FHSXT’ was accompanied by a reduction in branch number. SLs play an important role in regulating the outgrowth of a lateral bud. Therefore, the transcript levels of genes involved in SLs biosynthesis, including DWARF27 (D27), more axillary branches 3 (MAX3), more axillary branches 4 (MAX4), Cytochrome P450 family 711 subfamily A1 (CYP711A1) and Lateral branching oxidoreductase (LBO1), were analysed (Figure 7a). *MAX4* and *LBO1* showed a higher expression level in ‘FHSXT’ than in ‘QMH’. *MAX4* was 5.3‐fold higher in ‘FHSXT’ (Figure 7b). This result implied that the SLs contents may be higher in ‘FHSXT’ than in ‘QMH’.

**Figure 8:**
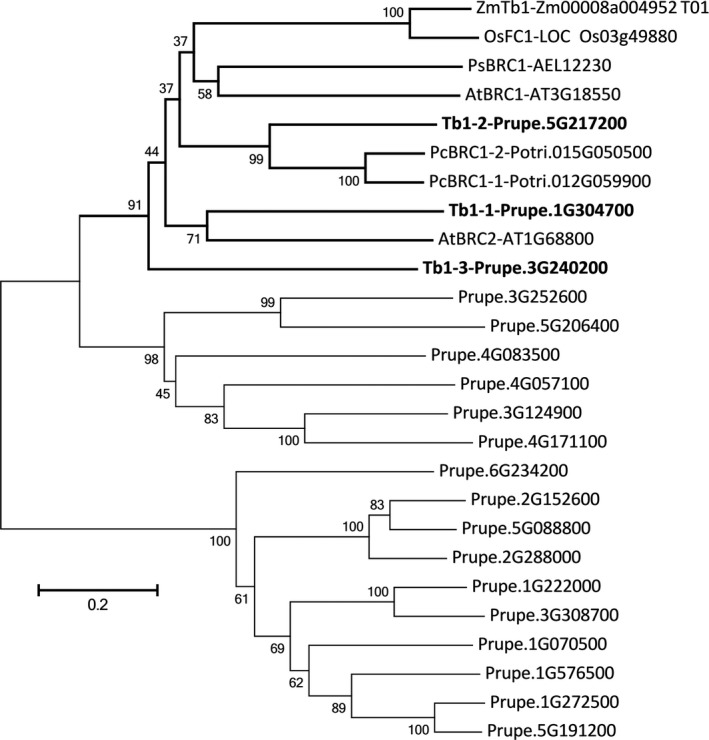
Phylogenetic tree of TCP transcriptional factor genes in peach genome and orthologous genes of *Tb1* from other species, including pea (PsBRC1, Braun *et al*., 2012), Arabidopsis (AtBRC1 and AtBRC2, Aguilar‐Martínez *et al*., 2007), poplar (PcBRC1‐1 and PcBRC1‐2, Muhr *et al*., 2016), maize (ZmTb1, Doebley *et al*., 1995), rice (OsFC1, Minakuchi *et al*., 2010). GenBank accession numbers are listed after the gene name. The orthologous genes of *Tb1* identified in peach are in bold. The numbers indicate bootstrap values calculated from 1000 replicate analyses.

**Figure 9:**
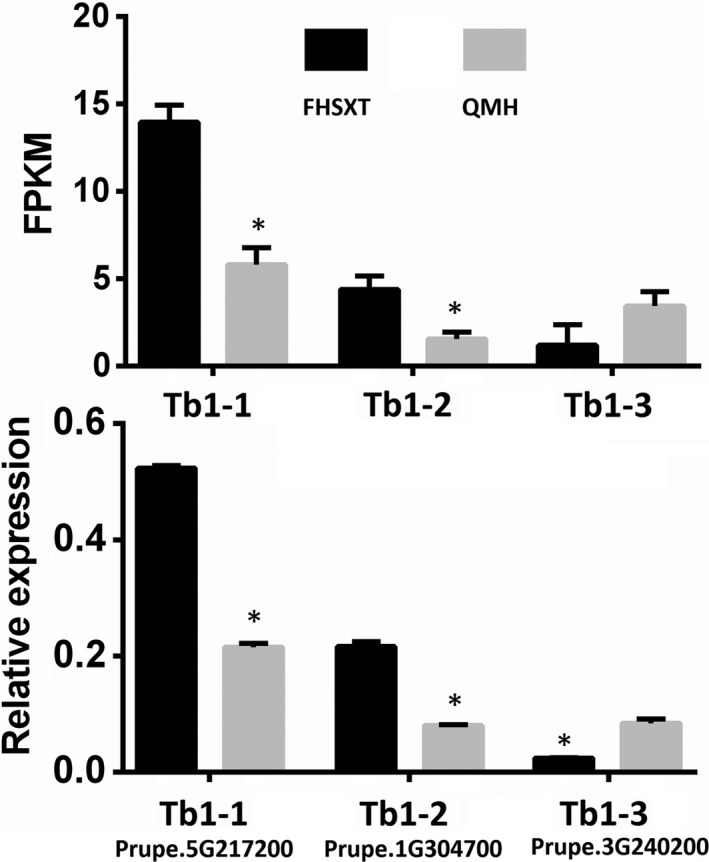
The FPKM and relative transcript levels of peach *Tb1* genes in shoot tips. The values represent the average of three biological replicates and error bars represent the standard deviation. * indicate significant difference at *P *<* *0.05 btween the two cultivars as determined by Student's *t* test.

Teosinte branched1 (Tb1) belong to the TCP transcription factor family and depresses the outgrowth of branch. The peach genome was searched for members of the TCP gene family, and 19 TCP transcription factors were identified. A phylogenetic tree composed of TCP transcription factors from peach and Tb1 from other species showed that all Tb1 transcription factor from other species and three peach TCPs closely clustered together. This suggested that there are three Tb1 orthologous genes in peach, including Prupe.5G217200 (Tb1‐1), Prupe.1G304700 (Tb1‐2) and Prupe.3G240200 (Tb1‐3; Figure 8). Analysis of qPCR and transcriptome data showed that the transcript level of Tb1‐1 and Tb1‐2 was significantly higher in ‘FHSXT’ than in ‘QMH’ (Figure 9). Tb1‐3 was down‐regulated significantly in ‘FHSXT’ according to the qPCR result.

## Discussion

Fining peach varieties with dwarfing traits is an area of intense interest due to the tendency of using dwarfed fruit trees in commercial orchards. Up to now, four kinds of dwarf traits have been identified in peach. Brachytic dwarfism is determined by *Dw* or *Dw2* loci and the dw/dw or dw2/dw2 homozygote presents with short internodes, thickened stems, reduced higher order branching, elongated leaves and normal fruit (Hansche, 1998). Homozygous individuals for *dw3* presents narrow branches and willowy growth (Chaparro *et al*., 1994). The ‘A72’ semidwarf peach with a forked branching character is an *N/n* heterozygotes, while the *n/n* homozygote has dwarf traits (Hu and Scorza, 2009). The forth type of dwarf traits is determined by the temperature‐sensitive semi‐dwarf (Tssd) locus, which depresses the internode growth below 30 °C (Lu *et al*., 2016). In this study, ‘FHSXT’ presents short internodes, reduced branching and elongated leaves. This suggested that the dwarf trait of ‘FHSXT’ is a type of brachytic dwarfism and its forked branching character is genetically different to that of ‘A72’ (Hu and Scorza, 2009). Brachytic dwarfism in peach trees has been reported to be caused by a non‐sense mutation within *PpeGID1c* (Hollender *et al*., 2016). In this study, a mutation in *GID1c* was identified in the cultivar ‘FHSXT’, and its molecular mechanism underlying the dwarf phenotype was illustrated.

### A nonsynonymous SNP in the *GID1c* coding sequence blocks the GA signalling pathway in ‘FHSXT’

Plant stature is mainly controlled by two factors, the number and length of internodes. In this study, the internode length of ‘FHSXT’ was significantly shorter than that of ‘QMH’. The number of internode was not analysed in these two cultivars, because they were difficult to count due to the luxurious growth of the peach tree. However, it has been suggested that brachytic dwarfism in peach is primarily attributable to reduced internode length (Hollender *et al*., 2016). Thus, we suggest that the reduced internode length was the major determinant of the dwarf trait of ‘FHSXT’. Observation of the paraffin sections demonstrated that reduced cell length is the main cause of the reduced internode length of ‘FHSXT’ (Figure 1). This suggested that the mutated gene(s) responsible for the dwarfism of ‘FHSXT’ inhibits cell elongation during the growth of shoots. GA is one plant hormone crucial for positive regulation of plant stature through regulating cell elongation (Gao *et al*., 2016; Zhou *et al*., 2016). These findings implied that the mutation(s) in ‘FHSXT’ may occur in the GA biosynthesis or signalling pathway.

GAs have multiple biological functions, especially in promoting stem elongation (Bömke and Tudzynski, 2009). Mutations in GA biosynthesis genes (GA‐sensitive mutant) and GA signal pathway (GA‐insensitive mutants) do change plant stature (Fambrini *et al*., 2011; Yamaguchi, 2008). Our results strongly demonstrated that ‘FHSXT’ is a GA‐insensitive mutant and its dwarfed phenotype is not due to GA deficiency. Interestingly, the dwarfing cultivar ‘FHSXT’ had a mass of endogenous GAs. This contradictory phenomenon of a dwarf trait accompanied by high accumulation of GAs was also reported in GA‐insensitive mutants of model plants and is caused by the DELLA‐dependent feedback regulation of GA biosynthesis. The DELLA proteins are master components of GA signalling, are repressors of plant growth, and are degraded after binding to GAs (Sun, 2010). On the other hands, DELLA proteins promote the expression of GA synthesis genes (Fukazawa *et al*., 2014, 2017 and Weston *et al*., 2008). The DELLA‐dependent feedback regulation of GA biosynthesis has been verified in many GA‐sensitive and ‐insensitive mutants. The Arabidopsis and rice F‐Box protein Sleepy1/GID1 targets DELLA protein for its gibberellin‐induced degradation. Compared with wild type, the dwarf mutant *sleepy1*/*gid2* showed no difference in *RGA* transcript level but highly accumulates RGA/SLR1 protein and the transcripts of *GA3ox* in Arabidopsis and *GA20ox* in rice (Dill *et al*., 2004; McGinnis *et al*., 2003). The *gid1* mutant in rice and Arabidopsis showed severe dwarf phenotypes and contained excessive amounts of DELLA protein and significantly higher concentration of endogenous GAs compared with wild type (Griffiths *et al*., 2006; Ueguchi‐Tanaka *et al*., 2005). In tomato, overexpressing *rgaΔ17* (RGA lacking the DELLA domain which is not degraded by exogenous GAs) highly up‐regulates the transcript level of *GA20ox* and has a dwarfed phenotype (Livne *et al*., 2015). In pea, mutation of DELLA proteins LA and CRY significantly depresses the transcript levels of the GA synthesis genes *GA20ox* and *GA3ox* (Weston *et al*., 2008). Recently, it has been demonstrated that DELLA can positively regulate the transcription of *GA20ox* by interacting with GAF1 (Fukazawa *et al*., 2014, 2017). Therefore, it was speculated that DELLA proteins are highly accumulated in ‘FHSXT’, which then represses the stem elongation and induces the expression of GA biosynthetic genes. Western Blotting showed that ‘FHSXT’ contained a higher amount of DELLA protein than ‘QMH’ (Figure 6). This result strongly suggested that the highly accumulated DELLA protein is responsible for the dwarf phenotype and high level of GAs in ‘FHSXT’. While a defect in the GA biosynthesis or signalling pathway can induce accumulation of DELLA, the first can be excluded because of the high content of endogenous GAs in the shoot tip of ‘FHSXT’.

The GA signalling pathway has three crucial components, namely GID1, GID2 and DELLA (Sun, 2010). The rice/Arabidopsis gid1 or gid2 mutants accumulate a high level of DELLA protein (Dill *et al*., 2004; Griffiths *et al*., 2006; McGinnis *et al*., 2003; Ueguchi‐Tanaka *et al*., 2005). DELLA protein accumulation is also induced when the mutation is located at DELLA or VHYNP motif of DELLA protein (Willige *et al*., 2007). Therefore, the orthologous *GID1*,* GID2* and *DELLA* genes was identified in the peach genome. No difference in the coding sequences of *GID2* and *DELLA* were identified between ‘FHSXT’ and ‘QMH’. Two *GID1* genes, including *GID1c* and *GID1b*, were identified in the peach genome, with GID1c already identified as positively regulating the stature of peach tree (Hollender *et al*., 2016). A nonsynonymous SNP from C to T was identified in the *GID1c* in ‘FHSXT’, which resulted in a new allele *GID1c* ^*S191F*^. Our result showed that the allele *GID1c* ^*S191F*^ has a close relationship with a dwarf phenotype in peach (Figure 4a). The same C to T mutation in *GID1c* was reported to cosegregate with brachytic dwarfism in offspring from an open pollination of ‘Nectavantop’ cultivar (Cantín *et al*., 2018). These results suggested that a single base pair mutation from C to T in the coding sequence of *GID1c* is closely correlated with the dwarf phenotype of peach.

GID1 receptors evolves from a larger family of Hormone Sensitive Lipases (HSLs) and several conserved motifs, such as HGGG and GDSSG motif, are retained to form the GA binding pocket (Gazara *et al*., 2018). The last two residues of the GDSSG motif are essential for the GID1–DELLA interaction (Ueguchi‐Tanaka *et al*., 2007). Alignment of GID1 from different species showed that Ser‐191‐Phe conversion occurred in the conserved motif GDSSG (Figure 4b). This suggested that GID1c^S191F^ would be unable to bind GA or interact with DELLA. In this study, interaction analysis of GID1c‐DELLA and GID1c^S191F^‐DELLA showed that GID1c^S191F^ was unable to interact with the DELLA protein even in the presence of GA (Figure 5). These results suggested that the GID1c^S191F^ blocks the GA signalling pathway, resulting in the accumulation of DELLA protein which then causes dwarfed trait of ‘FHSXT’ and accumulation of GAs.

Since GID1 physically interacts with DELLA apart from GA (Sun, 2010), it is likely that more conserved motifs are needed for these dual roles. In rice, mutations of conserved amino acid in GID1 would abolish its function. The conversion of G169E (*gid1‐5*), G196D (*gid1‐1*) or R251T (*gid1‐2*) in GID1 causes severe dwarf phenotypes (Ueguchi‐Tanaka *et al*., 2005, 2007). Deletion of E343 (*gid1‐7*) and conversion of L45F (*gid1‐8*) causes a milder phenotype (Ueguchi‐Tanaka *et al*., 2007). All these results suggest that single amino acids in conserved motifs are indispensable for GID1 function. Interestiongly, conversion of P99S in *gid1‐8* rescued its dwarf phenotype (Yamamoto *et al*., 2010). These results imply that analysing the function of conserved amino acids in GID1 might lead to precise gene editing targets that would regulate plant stature.

### GA signal pathway may regulate the lateral branch number by regulating the biosynthesis of SLs

SL, auxin and cytokinin (CK) are three key phytohormones that coordinately regulate shoot branching (Ni *et al*., 2015). Lateral bud outgrowth is repressed by auxin and SL, but induced by CK. SL acts downstream of auxin to depress bud outgrowth in pea and Arabidopsis, and GR24 (an analog of SL) efficiently suppresses the lateral bud outgrowth induced by decapitation or CK treatment (Brewer *et al*., 2009; Dun *et al*., 2012). It is suggested that SL plays a vital role in depressing the outgrowth of lateral bud. Strigolactone is biosynthesized in both the roots and shoots (Foo *et al*., 2001), where it promotes and represses the growth of lateral roots and lateral branches, respectively (Smith, 2013). In our study, transcripts of the strigolactone biosynthesis genes Max4 and LBO were significantly higher in shoots of ‘FHSXT’. This suggested that ‘FHSXT’ synthesizes more SL than ‘QMH’, which depressed the outgrowth of lateral buds. The level of SL was not determined in this study because SLs are difficult to quantify as a result of their low concentration and high instability (Akiyama and Hayashi, 2006). However, the SL‐regulated genes were analysed. Teosinte branched1 (Tb1), a TCP transcription factor, is a key component in the SL signalling pathway and plays a role in repressing the growth of lateral branches in Arabidopsis, pea, poplar and rice (Braun *et al*., 2012; Finlayson *et al*., 2010; Minakuchi *et al*., 2010; Muhr *et al*., 2016). Three orthologous genes of Tb1 were identified in the peach genome (Figure 8). Tb1‐1 and Tb1‐2 were more highly expressed in ‘FHSXT’ than ‘QMH’. Tb1‐3 was highly expressed in ‘QMH’. This indicated that perhaps Tb1‐3 is not invoved in the SL signalling pathway. This result is similar to that observed in poplar. Four orthologous *Tb1* genes were isolated in the poplar genome, but only two *Tb1* genes (*Potri.012G059900* and *Potri.015G050500*) are components of SL signalling (Muhr *et al*., 2016). All these results suggested that the accumulation of SLs in ‘FHSXT’ activates the transcription of Tb1, which then depresses the outgrowth of lateral bud.

Recent research showed that GA_3_ treatment reduces the levels of SL in rice and *Lotus japonicus*, while endogenous SLs levels are high accumulated in the DELLA‐accumulating mutant *gid2* and undetectable in the DELLA‐lacking mutant *slr1‐5* (Ito *et al*., 2017; Marzec, 2017). In this study, GID1c^S191F^ lost the ability to interact with DELLA1 protein, and resulting in a higher level of DELLA1 proteins in ‘FHSXT’ compared with ‘QMH’. This result suggested that the higher transcript level of the SL biosynthesis genes *Max4* and *LBO* detected in ‘FHSXT’ were caused by the high accumulation of DELLA1, which results in the accumulation of SLs. Our study suggested that blocked GA signalling in peach depresses the outgrowth of lateral bud. However, there are conflicting views on the role GA plays during the outgrowth of lateral bud. Rice and switchgrass overexpressing *GA2ox* exhibited dwarfism and increased tillering, and treatment with 5 mm of biologically active GA (GA3) represses tillering in wild‐type (Lo *et al*., 2008; Wuddineh *et al*., 2015). Mutation in the GA signalling pathway induced the outgrowth of branches from the rosette node in Arabidopsis (Bennett *et al*., 2016; McGinnis *et al*., 2003). These results suggested that GA plays a role in repressing the outgrowth of lateral bud. However, poplar overexpressing *PtaGA2ox1* exhibited dwarfism but reduced branch number (Busov *et al*., 2003). Gibberellin promotes shoot branching in the perennial woody plant *Jatropha curcas* (Ni *et al*., 2015). Our study also showed that the blocked GA pathway results in the reduction in sylleptic branch number in peach. Analysing all these results, we speculated that GA reduces the lateral branch number of herbaceous plants such as Arabidopsis, rice and switchgrass, but promote the outgrowth of lateral buds in woody plants such as poplar, *J. curcas* and peach. This speculation, possibly related to the degree of plant lignification, needs to be confirmed by further experiments.

**Figure 10:**
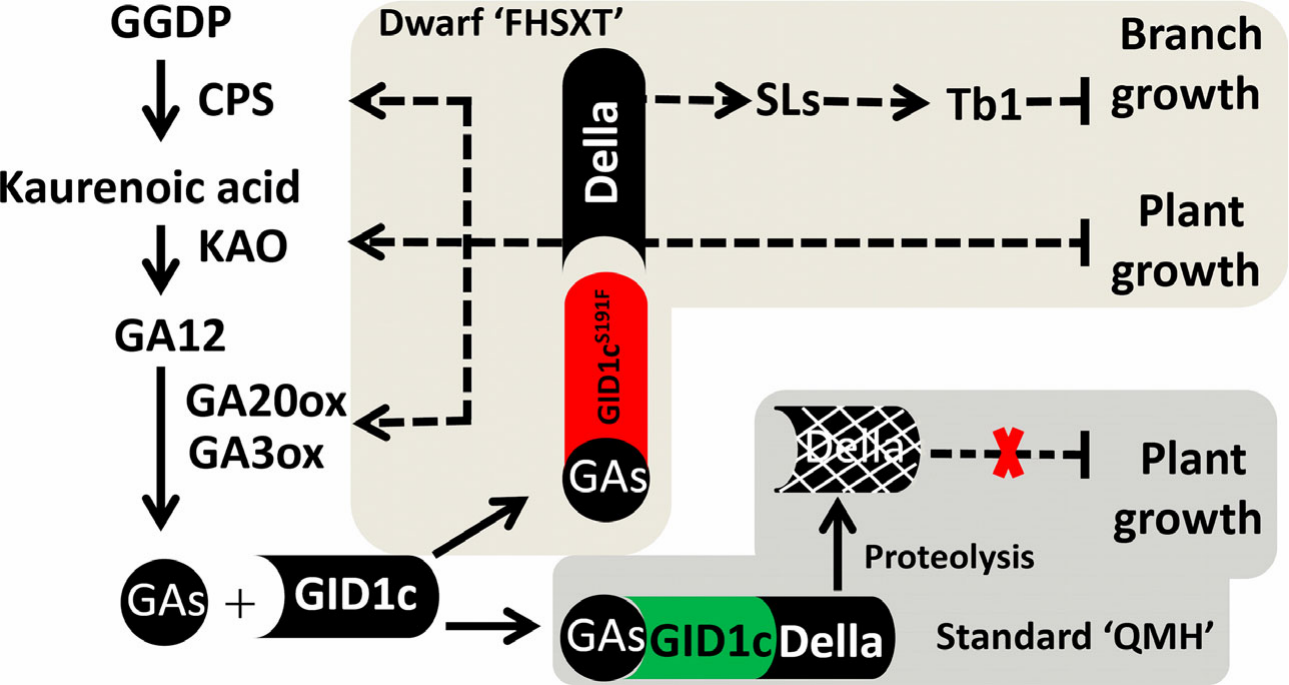
A model of the underlying mechanism influencing growth and branching phenotypes of ‘FHSXT’ and ‘QMH’.

We propose a model of the regulatory mechanism underlying peach tree architecture (Figure 10). In a standard cultivar like ‘QMH’, GID1c interacts with DELLA in the presence of GAs, inducing the degradation of DELLA, which then relieves the repression of DELLA on growth. In ‘FHSXT’, GID1c^S191F^ is unable to interact with DELLA protein, which results in the high accumulation of DELLA. A high level of DELLA simultaneously activates the transcription of GA biosynthesis genes and continues to repress elongation of cells within the stem, resulting in shorter internodes and thus entire trees of shorter stature; In other hand, high level of DELLA leads to the accumulation of strigolactone, activating the transcription of Tb1, which then depresses the outgrowth of lateral branches. These results show that GID1c can be targeted as a master regulator of peach tree architecture for future genetic manipulation.

## Materials and methods

### Plant material

Thirty peach accessions were used in this study that represent four kinds of growth habits (dwarf, standard, pillar and weeping; Table S1). These accessions are maintained at the Fruit Tree Germplasm Repository of Henan Agricultural University (Henan Province, China). All samples were prepared in the spring of 2017 from 2‐year old trees. The shoot tips with no leaves were collected from first or secondary branches and used for hormone quantification and RNA‐seq. Young leaves was used for genomic DNA extraction. Three samples from different trees for each cultivar were used as biological replicates. All samples were immediately frozen in liquid nitrogen, and then stored at −80 °C until use.

### Paraffin section

The unlignified and semi‐lignified stems were fixed in formaldehyde: glacial acetic acid: 70% ethanol (1 : 1 : 18 by volume), and dehydrated in a gradient ethanol series. The samples were embedded in Paraffin. Sections cut to 3‐μm thickness were applied to silane‐coated glass slides and the paraffin was removed from the sections. The sections were dehydrated through a gradient ethanol series, and then stained with fast green and the counterstain safranin before observation on a scanning electron microscope.

### Hormone treatment

Two‐year‐old trees were used to investigate DELLA protein levels in ‘FHSXT’ and ‘QMH’ after GA_3_ or H_2_O treatment. Three peach trees from each cultivar were sprayed with a 150 mg/L solution of GA_3_. Three hours after spraying, shoot tips were collected and frozen in liquid nitrogen. A set of peach trees from each cultivar was sprayed with water to serve as normal condition.

To investigate the growth of branches after GA_3_ treatment, five peach trees from ‘FHSXT’ and ‘QMH’ were sprayed every 3 d between 30 May 2017 and 13 June 2017 with a 150 mg/L solution of GA_3_. An identical set of plants was sprayed with water to serve as control.

### RNA‐Seq analysis

Total RNA was extracted using the ZP401 kit (Zoman, Beijing, China) according to the manufacturer's instructions. Three biological replicates for each cultivar were made into six cDNA libraries using the RNA Library Prep Kit according to the manufacturer's instructions (NEB, Beijing) and sequenced on the BGISEQ‐500 Platform. Before bioinformatics analyses, reads of low‐quality or containing adaptor sequence and high content of unknown bass (N) were removed. The filtered reads were then aligned to the peach genome (https://phytozome.jgi.doe.gov/pz/portal.html#!info?alias=Org_Ppersica) using Tophat (Trapnell *et al*., 2009). Following alignment, the count of mapped reads from each sample was derived and the gene expression level was calculated with RSEM (Li and Dewey, 2011). Transcript abundance was estimated from the number of Fragments Per Kilobase of transcript per Million mapped reads (FPKM). Differentially expression genes (DEGs) between ‘FHSXT’ and ‘QMH’ were detected with DEseq2 (Love *et al*., 2014). Raw P values were adjusted for multiple testing using a false discovery rate (FDR; Benjamini and Hochberg, 1995). Genes with a FDR of less than 0.05 and fold‐changes >2 were regarded as DEGs. Hierarchical clustering for DEGs was performed using pheatmap (REF).

### Quantitative real‐time PCR

Total RNA was extracted using the ZP401 kit (Zoman, Beijing, China) according to the manufacturer's instructions. Total RNA was treated with DNase I (Takara, Dalian, China) to remove any contaminating genomic DNA. Approximately 3 μg of total RNA was used for cDNA synthesis using PrimeScriptTM RT‐PCR Kit (Takara, Dalian, China). A SYBR green‐based real‐time PCR assay was carried out in 20 μl reaction mixtures containing 10.0 μL of 2 × SYBR Green I Master Mix (Takara, Dalian, China), 0.2 μm of each primer, and 100 ng of template cDNA. The peach actin gene PpGAPDH (Prupe.8G132000) was used as a constitutive control. Primer sequences for real‐time PCR are listed in Table S2. Amplification was conducted using StepOnePlus Real‐Time PCR System (Applied Biosystems, Foster, CA). The amplification program consisted of an initial denaturing step at 95 °C for 30 s, followed by 40 cycles of 95 °C for 30 s and 60 °C for 34 s. The fluorescent product was detected at the second step of each cycle. Melt curve analysis was performed at the end of 40 cycles to ensure the proper amplification of target fragments. Fluorescence readings were consecutively collected during the melting process from 60 to 90 °C at a heating rate of 0.5 °C/s. All analyses were repeated three times using biological replicates.

### Hormone quantification

Independent duplicate samples of shoot tips from ‘FHSXT’ and ‘QMH’ were analysed for GA content. Approximately 1 g of tissue was ground in liquid nitrogen and then added to 10 mL of extraction solution (Isopropanol/hydrochloric acid mixture). The mixture was shaken for 30 min at 4 °C. Dichloromethane (20 mL) was added to the mixture before shaking for 30 min at 4 °C. The mixture was separated to two layers after centrifugation at 10 000 ***g*** for 5 min at 4 °C. The lower layer was collected and dried under N_2_, then dissolved in 400 μL methanol containing 0.1% formic acid. The extracts were filtered through a 0.22 μm Millipore membranes, and analysed using a HPLC ESI‐MS/MS system (Agilent, America).

The analytical column was a poroshell 120 SB‐C18, 2.1 × 150 mm, with a particle size of 2.7 μm (Agilent, America). The analytical column was sequentially eluted using mobile phase A (methanol containing 0.1% formic acid) and mobile phase B (water containing 0.1% formic acid). The linear gradient for phase A was as follows: 0–2 min, 20%; 2–14 min, 20%–80%; 14–15 min, 80%; 15.1 min, 20%; and 15.1–20 min, 20%. Mass spectra were acquired in positive ion mode. The settings of the mass spectrometer were as follows: Curtain gas 15 psi; ionspray voltage, 4500 V; ionspray temperature, 400 °C. The GA components were quantified according to the standards of GA_1_, GA_3_ and GA_4_.

### GID1 sequence alignment

OsGID1 (LOC_Os05g33730.1) was used as the query sequence and the orthologous genes of GID1 were selected from different plants, including Arabidopsis, tomato, citrus, maize, grapevine, poplar and apple. The gene accession number in the Phytozome database (https://phytozome.jgi.doe.gov/pz/portal.html) are as follows: AT5G27320 (AtGID1c), AT3G05120 (AtGID1a), AT3G63010 (AtGID1b), SlGID1‐1 (Solyc01g098390),SlGID1‐2 (Solyc06g008870), CrGID1 (orange1.1g019235m), VvGID1 (GSVIVT01022014001), ZmGID1 (Zm00008a031335_T01), ProGID1 (Potri.013G028700), MdGID1 (MDP0000293827). Multiple sequence alignment was performed with Clustal W alignment.

### Yeast two‐hybrid system

The primers used for plasmid construction in this study are listed in Table S3. For analysing the interaction between GID1c and DELLA1 and 2, the full‐length cDNAs of GID1c and GID1c^S191F^ were cloned into the pGBKT7 vector (generating BD:GID1c and BD:GID1c^S191F^), while DELLA1 and DELLA2 was cloned into the pGADT7 vector (generating AD:DELLA1 and AD:DELLA2). Yeast two‐hybrid interaction studies were performed in the yeast strains Y2HGold. In brief, four pairs of constructs, namely BD:GID1c‐AD:DELLA1, BD:GID1c‐AD:DELLA2, BD:GID1c^S191F^‐AD:DELLA1, BD:GID1c^S191F^‐AD:DELLA2, were co‐transformed into Y2HGold. BD and AD plasmids were selected on dropout medium lacking Leu and Trp. The Y2H assay was performed on SD/‐Leu/‐Trp+X‐α‐Gal and ‐SD/‐Leu, ‐Trp, ‐Ade and ‐His in the absence and presence of 100 μm GA_3_.

### Antibodies and Immunoblotting Assay

Anti‐DELLA1 antibodies were generated in rabbits by immunizations with a synthetic peptide containing 12 amino acids (TCTDQNGSKGEY) from the N terminal of DELLA1. Anti‐DELLA1 antibodies were affinity purified. Peach shoot tips were ground in liquid nitrogen and the proteins were extracted using the Plant Total Protein Extraction Kit (Sangon, China). The protein concentration of the soluble fraction was determined using non‐interference protein assay kit (Sangon, China). For protein gel blot analysis, protein was separated by SDS‐PAGE and transferred to a PVDF membrane. The primary antibodies were used at a 1 : 4000 dilutions. The secondary antibodies (goat anti‐rabbit IgG coupled with horseradish peroxidase) were detected with the ECL system. Coomassie brilliant blue‐stained rubisco small subunit (RbcS) protein was used as a loading control.

### Identification of TCP transcription factor and Teosinte branched1 (Tb1) ortholog genes in peach

The Hidden Markov Model (HMM) profile of the TCP domain (PF03634) (http://pfam.sanger.ac.uk/) was then employed as a query to search the peach genome database (https://www.rosaceae.org/organism/Prunus/persica) using the program HMMER3.0 with the default *E*‐value. After determining the integrity of the TCP domain using the online program SMART (http://smart.embl-heidelberg.de/) with an *E*‐value < 0.1 and sequence alignment, 19 non‐redundant TCP genes were identified in the peach genome.

Phylogenetic trees were constructed using MEGA6.0. The neighbour‐joining (NJ) method was used to construct different trees with the pairwise deletion option. For statistical reliability, bootstrap analysis was carried out with 1000 replicates to evaluate the significance of each node.

## Conflict of interest

The authors declare no conflict of interest.

## Author contributions

JF, BT and JC conceived and designed the experiments; JC, MZ, YJ, ZG and TX performed the experiments; JF and JC wrote the paper; and XZ, XY, WW and JL revised the manuscript.

## Supporting information


**Figure S1** Representative leaves from 2‐year‐old ‘FHSXT’ (left) and ‘QMH’ (right) tree.
**Figure S2** (a) Hierarchical clustering and (b) heatmap of Pearson correlation between the six samples of shoot tips from ‘FHSXT’ and ‘QMH’.
**Figure S3** Sequence alignment of plant DELLA protein.
**Figure S4** Analysis of GID1c and GID1c^S191F^ interactions with DELLA2.
**Table S1** Cultivars used in this study and their growth habites.
**Table S2** List of primers used in this study.

**Table S3** The 2198 DEGs discovered in the transcriptomes of shoot tips from ‘FHSXT’ compared to ‘QMH’.
