# Supplementary material for: A single nucleotide mutation in GID1c disrupts its interaction with DELLA1 and causes a GA‐insensitive dwarf phenotype in peach
Source: Plant Biotechnol J. 2019 Mar 6;17(9):1723–35. doi: 10.1111/pbi.13094 (PMC6686139; doi:10.1111/pbi.13094)
Supplement: Supplementary file 1 — Figure S1 Representative leaves from 2‐year‐old ‘FHSXT’ (left) and ‘QMH’ (right) tree. Figure S2 (a) Hierarchical clustering and (b) heatmap of Pearson correlation between the six samples of shoot tips from ‘FHSXT’ and ‘QMH’. Figure S3 Sequence alignment of plant DELLA protein. Figure S4 Analysis of GID1c and GID1cS191F interactions with DELLA2. Table S1 Cultivars used in this study and their growth habites. Table S2 List of primers used in this study. [file PBI-17-1723-s002.docx]

**A single nucleotide mutation in *GID1c*** **disrupts its interaction with DELLA1 resulting in a GA-insensitive dwarf phenotype in peach**

Authors: Jun Cheng^#^, Mengmeng Zhang^#^, Bin Tan, Yajun Jiang, Xianbo Zheng, Xia Ye, Zijing Guo, Tingting Xiong, Wei Wang, Jidong Li, Jiancan Feng^*^

The following Supporting Information is available for this article:

**
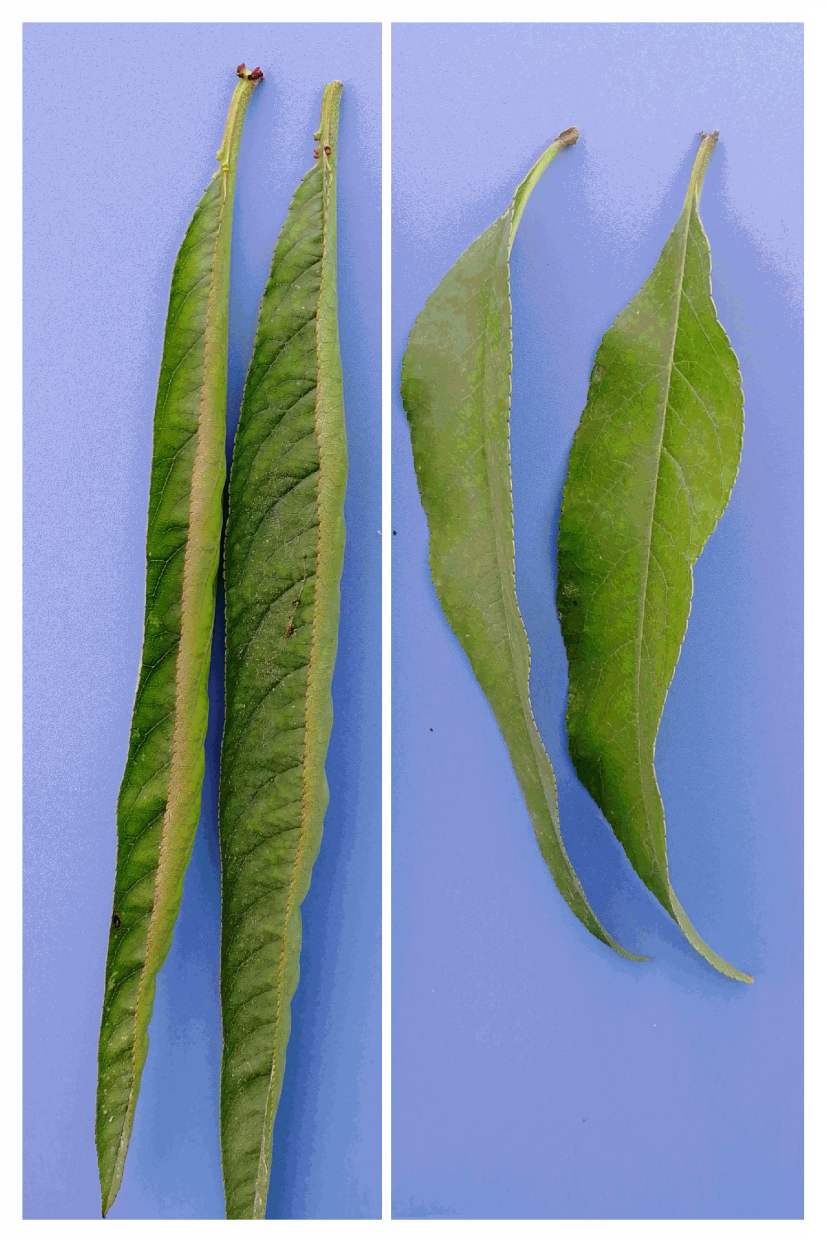
**

**Fig. S1** The length of leave from ‘FHSXT’ (left) and ‘QMH’ (right)

**
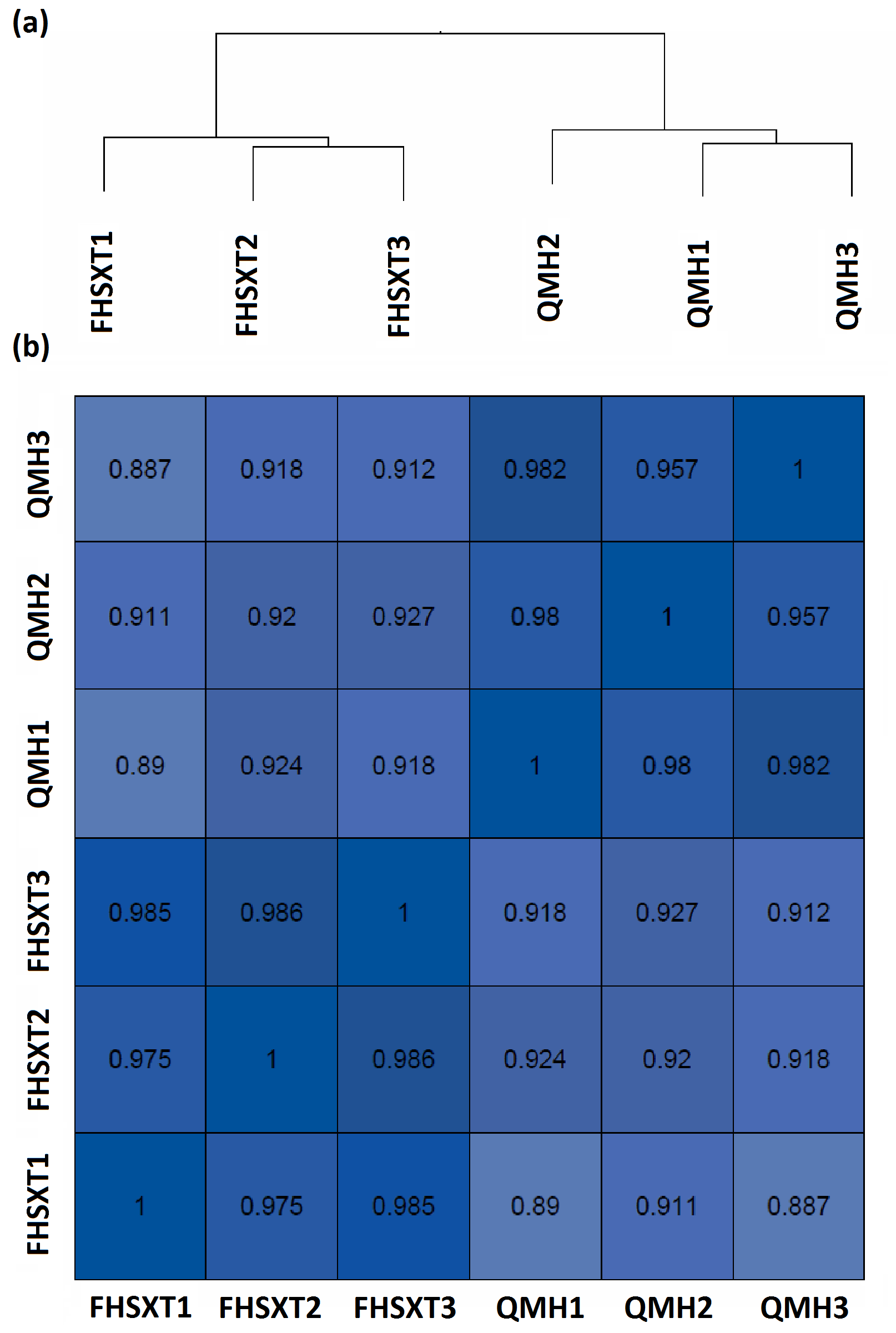
**

**Fig. S2** (a) Hierarchical clustering between samples and (b) heatmap of Pearson correlation between samples.

**
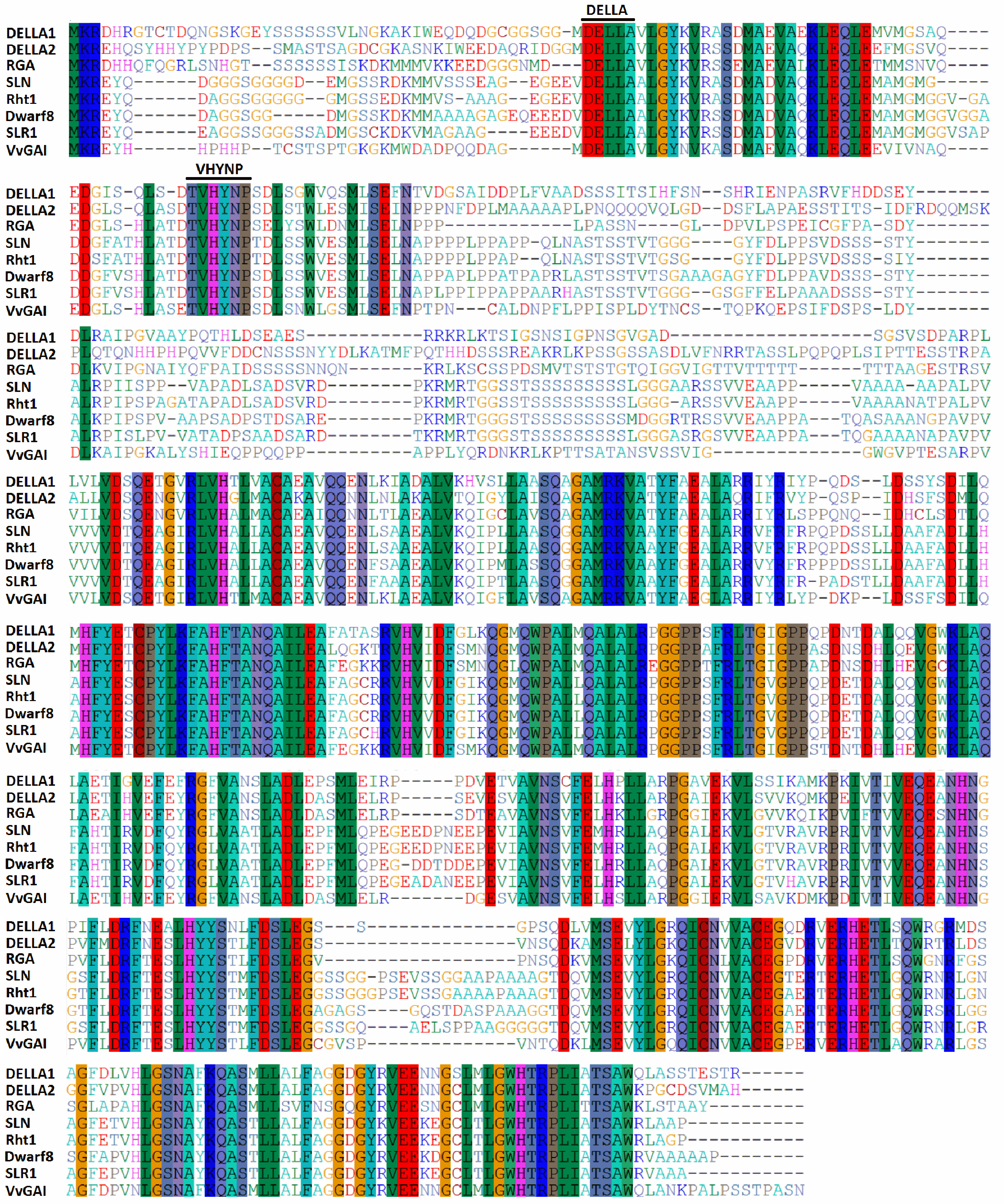
**

**Fig. S3** Sequence alignment of DELLA protein. The DELLA and VHYNP domain were highlighted. The amino acid sequences were downloaded from NCBI and its accesion number is: RGA (Q9SLH3), Rht1 (Q9ST59), SLR1 (Q7G7J6) and VvGAI1 (Q8S4W7).

**
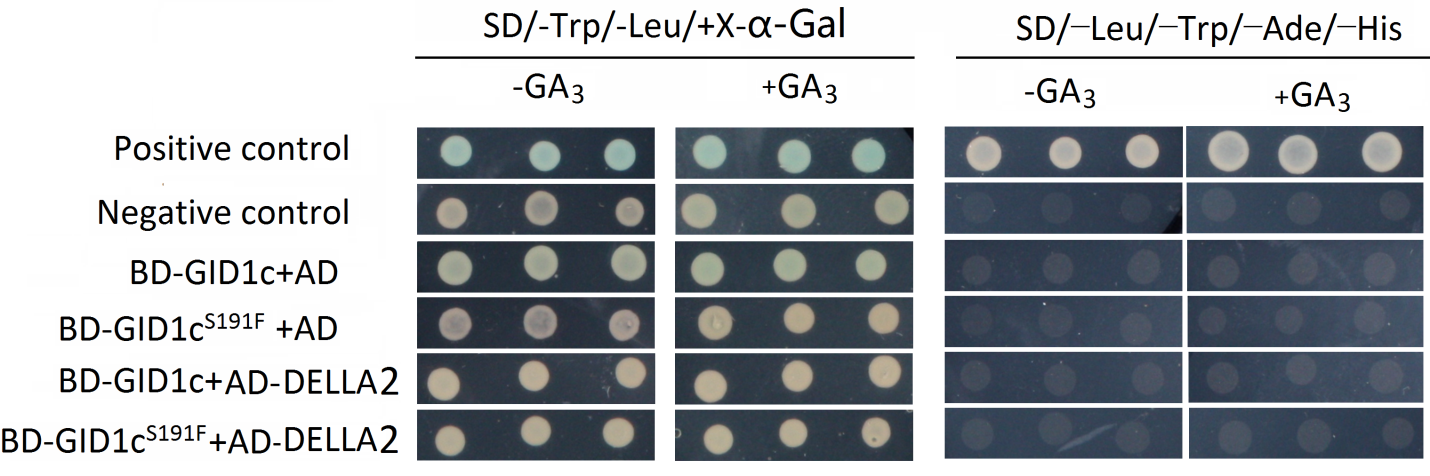
**

**Fig. S4** The activity analysis of GID1c and GID1c^S191F^ interacting with DELLA2. Y2H assay using full-length GID1c and GID1c^S191F^ as baits and the full-length SLR1 as prey in the presence and absence of 10^-4^ M GA_3_. The Y2H assay was performed on SD/-Trp/-Leu/X-α-Gal and Top: SD/-Trp/-Leu/-Ade/-His.

**Table S1**  Cultivars used in this study and its growth habites

| Cultivar | Growth habit | Cultivar | Growth habit |
| --- | --- | --- | --- |
| ‘Zhongai45’  ‘Ailihong’  ‘Ailimi’  ‘Shoubai’  ‘Shoufeng’  ‘Danshouhong’  ‘Danshoufeng’  ‘Baishouxing’  ‘Hongshouxing’  ‘Zhongai33’  ‘Q37’  ‘FHSXT’  ‘Ke+Shi’  ‘ZhongYou2’  ‘HuangJinMeiLi’ | Dwarf  Dwarf  Dwarf  Dwarf  Dwarf  Dwarf  Dwarf  Dwarf  Dwarf  Dwarf  Dwarf  Dwarf  Standard  Standard  Standard | ‘ZhongPan’  ‘TeZaoHong’  ‘ChunMi’  ‘ChunMei’  ‘YuMeiRen’  ‘QiuSuo’  ‘QiuTian’  ‘JinHui’  ‘HongCuiZhi’  ‘Zhaoshoubai’  ‘Dajiubao’  ‘Zhaoshoufeng’  ‘18Hao’  ‘Sahonglongzu’  ‘Zhaoshouhong’ | Standard  Standard  Standard  Standard  Standard  Standard  Standard  Standard  Weeping  Pillar  Pillar  Pillar  Pillar  Pillar  Pillar |

**Table S** **2**. List of primers used in this study.

| No. | Primer | sequence |
| --- | --- | --- |
| Primers for Real time PCR | | |
| 1 | CPS | 5’-CCTTCGGACGACATACACTCTT-3’/5’-GCACTGTGGCTAATCCTATGG-3’ |
| 2 | KS | 5’-ACAGGGTCGTCACTGAGGAGG-3’/ 5’-GGCAACGATGGGTTCTTCAG-3’ |
| 3 | KO | 5’-CAATGGGAAAGCCCTGGAG-3’/ 5’-CTGAAGAGAACCAGCACAAACC-3’ |
| 4 | KAO | 5’-GGCAAGAATCAAGAAAACTGGA-3’/5’-GCCAACCAAAAACATAGAACCT-3’ |
| 5 | GA20ox1 | 5’-AACAGCCAAACACCAAGGAAGT-3’/5’-TCTTGGGCACGAAGTATCATC-3’ |
| 6 | GA20ox2 | 5’-TGATTCCAAGAGCCCAAGG-3’/5’-ATCCAGTCTGAGAAGGCATCC-3’ |
| 7 | GA20ox3 | 5’-CAGAAGGAGAGGAGGTCAATGG-3’/ 5’-CTTTTGGAGGGTAGTTTCGTCAG-3’ |
| 8 | GA3ox | 5’-CCCTCCTCACAATCCTTTACC-3’ /5’-GATGGATAAACGGTGTCTGCTC-3’ |
| 9 | GA2ox1 | 5’-GCATAGGGTTTTGGCAGACAC-3’/5’-GGACTTTTTGTATTCACACCACG-3’ |
| 10 | GA2ox2 | 5’- TTTGATTGGTGAGGGATTGTG-3’/5’- GCTACTGTTGATGTTGCTGGTG-3’ |
| 11 | GID1c | 5’-AGGGGAAGACAGGGACCAC-3’ /5’-AAACCAGCCACCACGACAA-3’ |
| 12 | GID1b | 5’-TGAAGCAGAAGTTGAGGTATTGG-3’/ 5’-CTGGGTGGTCTCTGTCTTCTCC-3’ |
| 13 | Della1 | 5’-ACAGGGTGGAGGAGAACAATG-3’/ 5’-CAAGGGTCTTCAGAGGTCAGTT-3’ |
| 14 | Della2 | 5’-TGAACAGTCAGGATAAGGCGAT-3’/ 5’-GGCAAACAAAGCCAACAGC-3’ |
| 15 | GID2-1 | 5’-TCTTCCTCATCGTCGTCAGC-3’/5’-CGAGAGAAGCGAGAGTGATAGAT-3’ |
| 16 | GID2-2 | 5’-ACAAGCGCCTCTACAGGGTCT-3’/ 5’-GCCGCTTCGCTCGTAGTAGT-3’ |
| 17 | D27-1 | 5’-TGGAGCCAAACTTCAGCGAC-3’/ 5’-TCTGCGTCGTGTGGCGTTA-3’ |
| 18 | D27-2 | 5’-GTGAAGCAAAGAAGTGGAGTCC-3’/ 5’-GAATCATGGTTAAGGGAAGCC-3’ |
| 19 | MAX3 | 5’-CCTCGGTCTCCTAATGAAAATG-3’/ 5’-GAGGTAGCGTCTTGCTTTGG-3’ |
| 20 | MAX4 | 5’-GATGGTTAGTGGGAAAAATGGA-3’/5’-AATCACTTTATTTGGAGGGAACC-3’ |
| 21 | CYP711A1 | 5’-AAAGAAGTGAAACAAAGGCATCC-3’/ 5’-GCTCCAGTTGTCTTGTTTCGTG-3’ |
| 22 | CYP711A2 | 5’-AGTTCGGCATTATTCTGGATTT-3’/ 5’-ACACAACAAAGACGCCACAAT-3’ |
| 23 | LBO1-1 | 5’-CTGTCACAACTGAAACCAAAGC-3’/ 5’-ATCTTGGCTGTTTCAATGTGTG-3’ |
| 24 | LBO1-2 | 5’-AAAAGAACGGCTGTCAATCG-3’/5’-TCTTGAACTCCCACCCTTTTAT-3’ |
| 25 | Tb1-1 | 5’-GGGGCTGGTTCATCACGAG-3’ /5’-GGCTCCACTTTCCCATAATCAC-3’ |
| 26 | Tb1-2 | 5’-CTCAGGCGGTCACTACTTATGG-3’ /5’-GCTGCCCACAACCTTCTCAT-3’ |
| 27 | TB1-3 | 5’-GGGCAAGGGAAAGGACAAG/GGAG-3’GGAGCCCAGTTCTTG-3’ |
| 28 | RP II | 5’-TGAAGCATACACCTATGATGATGAAG-3’/5’-CTTTGACAGCACCAGTAGATTCC-3’ |
| Primer for vector construction | | |
| 29  30 | GID1c  Della1 | 5’-TT***CATATG***ATGGCTGGGACCAACGAAGT-3’/5’-AA***GGATCC***ACCGCACGCGATGAAAGTC-3’  5’-CT***CATATG***ATGAAGAGAGATCACCGCGG-3’/ 5’-AG***CTCGAG***TCACCGGGTTGACTCAGTCG-3’ |
| 31 | Della2 | 5’-CT***CATATG***ATGAAAAGAGAGCATCAGAG-3’/ 5’-AG***CTCGAG***TCAGTGAGCCATCACCGAGT-3’ |
| Primer for detecting the SNP in GID1c | | |
| 32 | GID1c-SNP | 5’-TGACAAACCTGTGAGCAATGAG-3’/ 5’- CCTTCTCAAGCCCTTTAGCATA-3’ |
